# Convention of American Medical Colleges

**Published:** 1879-05

**Authors:** 


### CONVENTION OF AMERICAN MEDICAL COLLEGES.

ATLANTA, May 2d, 1879.

At 10½ o'clock the meeting was called to order by Dr. N. S. Davis, of Chicago, by whom Dr. S. D. Gross, of Philadelphia, was nominated for chairman and unanimously elected.

Dr. Starling Loving, of Columbus, Ohio, was unanimously elected Secretary.

Dr. Davis moved that a committee of three be appointed by the chair to receive the credentials of delegates to the convention.

Drs. Davis, Menees and Prioleau were appointed, who, upon examination of certificates, presented the following accredited delegates:

College Physicians and Surgeons, Baltimore—Faculty, Dr. E. Loyd Howard; Trustees, Dr. J. S. Lynch.

Miama Medical College, Cincinnati—Faculty, Dr. John A. Murphy.

Starling Medical College, Columbus, Ohio—Faculty and Trustees, Dr. Starling Loving.

State University of Iowa—Faculty, Dr. Thos. S. Parr; Trustees, Dr. W. F. Peck.

University of Michigan—Faculty, Dr. E. S. Dunster.

Kentucky School of Medicine—Faculty, Dr. H. B. Cook; Board of Regents, Dr. H. F. Calfers.

University of Louisiana—Faculty and Trustees, Dr. E. S. Lewis.

Womens' Medical College of Pennsylvania—Faculty, Dr. Frances Emily White; Trustees, Dr. C. N. Pence.

Rush Medical College—Faculty and Trustees, Dr. Moses Gunn.

Medical College of Ohio—Faculty and Trustees, Dr. W. W. Dawson.

Medical College State of South Carolina—Faculty, Dr. S. Ford Prioleau; Trustees, Dr. J. P. Chazal.

Detroit Medical College—Faculty, Dr. Laertes Connor.

Medical Department University of Louisville—Faculty, J. M. Boden; Trustees, Jno. E. Crowe.

Nashville and Vanderbilt University—Faculty, Dr. D. C. Kelly; Trustees, Dr. D. T. Menees.

Louisville Medical College—Faculty, Dr. C. N. Kelly; Trustees, Mr. W. B. Fleming.

Medical College of Indiana—Trustees, Dr. James F. Hibbard.

Atlanta Medical College—Faculty, Dr. J. G. Westmoreland; Trustees, Dr. J. T. Johnson.

Medical Department University of Maryland—Faculty, L. McLane Tiffany.

Jefferson Medical College—Faculty, Dr. Sam'l D. Gross.

Hospital College of Medicine, Louisville—Dr. Dudley S. Reynolds.

Chicago Medical College, Medical Department of the Northwestern University—Faculty, Dr. Nathan S. Davis.

Southern Medical College, Atlanta, Ga.—Trustees, Dr. T. S. Powell.

Medical College, Evansville, Ind.—Faculty, Dr. S. B. Walker; Trustees, Dr. H. G. Jones.

Dr. N. S. Davis, stating the object of the convention, read the resolutions as presented by Dr. S. D. Gross, and adopted by the American College Association, at Buffalo, N. Y., in 1878. The purport is to promote a close unanimity of colleges, and the discussion of the plan of having three annual courses of five or six months as necessary to graduation. He also suggested the plan of adding to the ordinary medical curriculum, studies in mathematics, natural history, etc.

Dr. Dunster, of Michigan, spoke of the instruction given him by his institution to support the three year plan. Remarked that the University of Michigan had determined to take this step, whether the Convention and Association resolved upon it or not.

Dr. N. S. Davis moved that a committee of five be appointed by the chair to draw up a plan of action for the Convention.

Dr. John A. Murphy, of Cincinnati, thought adversely to the motion of the gentleman from Chicago; could not understand the intention of the motion, and desired an explanation.

Dr. Tiffany, of Baltimore, spoke in favor of the motion to appoint the committee. He considered that the Convention was separate and distinct from the Association.

Dr. John S. Lynch, of Baltimore, favored the motion of Dr. Davis, on the grounds that it was the best means of forming a plan of proceeding independent of any connection with the College Association.

Dr. Hibbard made some remarks relative to the relation between the Convention and Association.

At this point the President ruled the discussion out of order.

Dr. Lynch amended the original motion by suggesting nine instead of five members of the committee.

Dr. Davis, while he thought it would render the committee more cumbersome, yet accepted the amendment.

Dr. Murphy pressed his desire of an explanation of the motive in wanting a committee to arrange affairs for discussion.

Dr. Davis in reply, spoke of the several ineffectual attempts that had been made by the American Medical College Association to elevate the standard of medical education. A convention of colleges being called, some of the schools answered, while some did not. To meet this lack of unanimity, certain schools which had not enlisted in the Association of Colleges, were called to co-opetate with the permanent body and form some plan to promote harmony, with the understanding that those not in the Association, should not be bound by the action of that body.

Asserted that he cared not if advancement of medical education came through the action of Association or Convention; so it was assured, and thought that the committee, as suggested, would not interfere with the Association.

Dr. J. G. Westmoreland of Atlanta, Ga., thought a convention of Colleges such as this seems to be, cannot have its action reviewed by the legislative body, the Medical College Association which meets to-morrow; conventions make supreme or fundamental law. Dr. Davis who seems to recognize this fact, at the same time declares that if certain measures are adopted by the Convention, he shall refuse to accept them as binding on the Association to-morrow, an inconsistancy requiring explanation.

Dr. Murphy did not think that the action of this Convention would have any effect with the Association.

Dr. Reynolds being sent with plenary powers, was willing to take the pledge of honor as suggested by the vote of this Convention, and thinks, that the committee proposed by Dr. Davis, should be permitted to proceed to the draughting a plan of business.

Dr. Bodine offered as amendment, that the committee include those delegates present that are not members of the Association of Colleges.

Dr. Menees favored the appointment of committee, or any plan that looked to the question of harmonizing discordant elements, by which unanimity in the attempt to advance medical education might be secured.

The resolution of Dr. Davis and the amendment of Dr. Bodine, were carried, and the chair appointed Drs. Davis, Chazal, Dunster, Tiffany and Reynolds: Delegates not in the Association, Drs. Westmoreland, Johnson, Powell, Frances Emily White, Lynch and Peck.

Dr. Peck moved that the committee as appointed, be required to report this afternoon at 3 o'clock, which being agreed to, the committee was so instructed by the chair.

On motion, the Convention adjourned till 3½ o'clock, P.M.

AFTERNOON SESSION.

The Convention was called to order by President, Dr. Gross.

The committee on credentials, reported the following additional delegates:

Nashville Medical College—Faculty, Dr. Dearing J. Roberts; Councilors, Dr. Duncan Eve.

Nashville University—Trustees, Judge A. L. Demans.

College of Physicians and Surgeons, Keokuk, Iowa—Faculty, Dr. J. C. Hughes.

These delegates were admitted on the recommendation of the committee as being duly accredited.

The Convention then proceeded to the consideration of the report of the committee appointed to draught some definite plan of business.

The report is as follows:

Your committee, after due deliberation, have decided to submit for the consideration of the Convention, only two propositions, and those without any expression of opinion concerning their merits by your committee.

The propositions are as follows:

*First*—That all Medical Colleges should require attendance upon three regular courses of lectures, during three separate years, before admitting students to become candidates for the degree of M.D.

*Second*—That all Medical Colleges should require, before admitting students to matriculation, a preliminary examination; such examination to embrace, at least, the elements of the Physical Science, in addition to a fair English education.

All of which is respectfully submitted,

N. S. Davis, Chairman.

Dr. Davis moved that the report be adopted.

Dr. Tiffanny moved as a substitute that the proposition be discussed seriatim. Carried.

Dr. Bodine offered the following resolution:

*Resolved*, That it is the opinion of this mass-meeting of American Medical Colleges, that the subject of medical education can best be entrusted to the American Medical College Association, and that the Convention do now adjourn *sine die*.

Upon agreement, the question as to the motion of Dr. Davis, and the resolution of Dr. Bodine, were held over for the purpose of discussing the merits of the proposition.

Dr. Davis spoke in support of the propositions.

Dr. Cook thought this Convention should work within its sphere, for the advancement of medical education, in connection with the Association of Colleges.

Dr. Dunster concluded, that so far as forestalling the action of the Association, as had been suggested, it will rather be benefited and facilitated by the discussions of the Convention. Objected very strongly to the resolution of Dr. Bodine.

Dr. Menees was in favor of a free and outspoken discussion of the issues in question by this Convention, but concluded that no definite action could be had unless there existed guarantees of adherence on the part of absent colleges.

Dr. Davis discussed at some length the merits of the Committee's report.

Dr. Bodine thought that this discussion amounted to nothing in the way of deciding the question. Desired to hear from delegates who are not members of the Association of Colleges.

The further discussion of the three year course project and the graded course plan was carried on by eloquent speeches from Drs. Davis, Murphy, Reynolds, Dunster, Gunn, Roberts, Howard, Thompson, Cook and Kelly.

Dr. Roberts offered the following resolution:

*Resolved,* That it is the sense of this Convention that a three years' course, of at least five months in each, of collegiate instruction, should be required for the degree of M.D., and that students entering college should have a good English education.

Dr. Howard thought it would be eminently unwise to adopt this resolution, because there are several delegates that are not so authorized to act.

Dr. Bodine thought it useless to pretend to assume any obligations in this Convention, as such could have no force or continuance.

The President, resigning the chair to Dr. Gunn, suggested that the Convention could only *recommend* these questions to the consideration of the Association—that there was no authority to assume any obligation.

The vote being taken on the adoption of proposition first, the ayes and nays being called by colleges, resulted in ayes twenty, nays four—two colleges not voting.

Dr. Davis moved that the second proposition be put, which was done, and passed.

Dr. Reynolds then moved that the whole subject be referred to the American Medical College Association, and that body be apprised of the action of this Convention. Adopted.

Convention adjourned *sine die.*

